# Fetuin-a to adiponectin ratio is a sensitive indicator for evaluating metabolic syndrome in the elderly

**DOI:** 10.1186/s12944-020-01251-5

**Published:** 2020-04-06

**Authors:** Zhongwei Zhou, Mingzhong Sun, Hao Jin, Hongmei Chen, Huixiang Ju

**Affiliations:** 1 Department of Clinical Laboratory, Affiliated Yancheng Hospital, School of Medicine, Southeast University, No. 75 Juchang Road, Tinghu, Yancheng, Jiangsu 224001 P.R. China; 2 Department of Blood Transfusion, Affiliated Yancheng Hospital, School of Medicine, Southeast University, Yancheng, Jiangsu 224001 P.R. China

**Keywords:** Fetuin-a, Adiponectin, Fetuin-a/adiponectin ratio, Metabolic syndrome

## Abstract

**Background:**

Fetuin-A and adiponectin present significant associations, supported by recent evidence, with metabolic syndrome (MS) featuring hyperglycemia, central obesity and insulin resistance as the main components, but their biological functions are opposite. The aim of this study was to verify whether fetuin-A/adiponectin ratio (F/A ratio) is a more sensitive indicator for evaluation of MS than either fetuin-A or adiponectin.

**Methods:**

In this cross-sectional study, 465 elderly subjects were selected from the physical examination database. Serum levels of fetuin-A and adiponectin were measured using an enzyme-linked immunosorbent assay (ELISA) method. Spearman’s rank correlation coefficient, linear regression and logistic regression analysis were adopted to estimate the correlations of fetuin-A, adiponectin and F/A ratio with MS and its components, and receiver operating characteristic (ROC) curve analysis was performed to evaluate the predictive values of the aforesaid indices.

**Results:**

Compared with fetuin-A or adiponectin, F/A ratio was significantly associated with all the components of MS, and this correlation was significant even after adjusting potential confounding factors (*P* < 0.05). Logistic regression analysis indicated that F/A ratio presented a stronger correlation with incident MS (adjusted OR: 1.466; 95% CI: 1.189–1.808) than fetuin-A (adjusted OR: 1.100; 95% CI: 1.020–1.186) and adiponectin (adjusted OR: 0.760; 95% CI: 0.664–0.871) alone. ROC analysis revealed that F/A ratio achieved a larger area under curve (AUC) than fetuin-A and adiponectin, with their AUC values of 0.755, 0.709 and 0.708, respectively.

**Conclusion:**

F/A ratio is a more sensitive index for evaluating MS than either fetuin-A or adiponectin in the elderly.

## Introduction

Metabolic syndrome (MS) is defined as a cluster of interrelated risk factors including hyperglycemia, hypertension, dyslipidemia, central obesity and insulin resistance, which associates with higher risk of suffering from type 2 diabetes mellitus (T2DM) and cardiovascular disease (CVD) [1]. Recently, economic modernization and fast-westernizing lifestyle have brought about the prevalence of MS in Chinese population, especially in the elderly, which has snowballed into a public health problem [2, 3]. Therefore, exploring an appropriate and effective screening marker for identifying individuals at high risks for MS is the preoccupation.

Fetuin-A, a protein mainly produced by liver cells and secreted into circulation at high concentrations, is an endogenous inhibitor of insulin receptor tyrosine kinase which results in insulin resistance [4]. The latest studies have uncovered that it is also secreted and expressed in adipose tissue, and this secretion is more opulent in visceral adipose tissue than in subcutaneous adipose tissue especially in the obese [5, 6]. Such is the way of how fetuin-A is involved in the development of obesity and insulin resistance. It is considered as a novel link between obesity and its complications such as MS [7]. Data from the Heart and Soul Study [8] has pointed out significantly positive correlations between increased fetuin-A levels and MS and between the levels and an atherogenic lipid profile in nondiabetic subjects with coronary artery disease. Further studies in middle-aged and elderly Chinese also have revealed significantly positive correlations between fetuin-A and MS and its components [9].

Adiponectin is one of adipokines that are almost exclusively secreted by adipocytes [10]. In contrast to fetuin-A, higher adiponectin levels are considered to be beneficial because of its antidiabetic and anti-atherogenic potential [11]. A large body of researches have demonstrated that low adiponectin levels may associate with risks and the severity of MS [12–14]. By contrast, increased adiponectin levels have been proven as an independent protector for the development and regression of MS in several prospective studies [15, 16].

Although fetuin-A and adiponectin are both intimately involved in MS, they act in opposite aspects. Previous genomewide scans have yielded a clear evidence that both fetuin-A and adiponectin genes are located at chromosome 3q27-qter which is a susceptibility locus for MS and early-onset diabetes [17]. Therefore, fetuin-A and adiponectin are speculated to work together in the metabolic balance, and fetuin-A/adiponectin ratio (F/A ratio) is expected to show more sensitive performance in assessing metabolic disorder than fetuin-A or adiponectin alone. As our previous study has uncovered the potential association of F/A ratio with MS and its components, which is stronger than either parameter alone [18], in this study we attempt to further evaluate F/A ratio as a promising predictor for determining MS in the elderly.

## Material and methods

### Study population

This cross-sectional study was conducted in the Affiliated Yancheng Hospital of Southeast University Medical College from March 2014 to March 2015. A total of 500 subjects aged 60 years or older who had health examinations were selected by a random number table. Thirty-five subjects with acute infectious disease, severe CVD, hepatic and renal dysfunction and malignant tumors were excluded from the study. Consequently, 465 elderly subjects were included in this study. The study protocol was approved by the Ethics Committee of the hospital and informed consent was obtained from all subjects involved in this project.

### Laboratory measurements

Body height and weight of all subjects were measured and then body mass index (BMI) was calculated as weight in kilograms divided by height in meters squared (kg/m^2^). Waist circumference (WC) was measured at the umbilicus level. Blood pressure was measured with the subject in a sitting position after a minimum 10-min rest.

Every participant’s blood sample was drawn in the morning after an overnight fast for at least 10 h. Levels of fasting plasma glucose (FPG), total cholesterol (TC), triglyceride (TG), high-density lipoprotein cholesterol (HDL-C) and low-density lipoprotein cholesterol (LDL-C) were determined using a biochemical autoanalyzer (Roche P800, Basel, Switzerland). Serum C-reactive protein (CRP) concentrations were detected by the rate nephelometry assay (IMMAGE 800, Beckman Coulter Inc. Brea, CA, USA). Insulin levels were quantitated by the electrochemiluminescence method, and homoeostasis model assessment of insulin resistance (HOMA-IR) index was calculated as follows: fasting insulin (uU/mL) × FPG (mmol/L) / 22.5. Serum levels of fetuin-A and adiponectin were determined by an ELISA method (Biovendor, Modrice, Brno, Czech Republic).

### Metabolic syndrome definition

According to the revised National Cholesterol Education Program Adult Treatment Panel III (NCEP-ATP III) criteria [19], MS must be diagnosed in the presence of 3 or more of the following 5 abnormalities: (i) a WC of ≥90 cm in men or ≥ 80 cm in women according to the criteria for South Asians/Asians; (ii) TG level ≥ 150 mg/dL (1.7 mmol/L) or receiving medications for increased TG; (iii) HDL-C level < 40 mg/dL (1.03 mmol/L) in men or < 50 mg/dL (1.3 mmol/L) in women, or taking medications for reduced HDL-C; (iv) systolic blood pressure (SBP) ≥ 130 mmHg or diastolic blood pressure (DBP) ≥ 85 mmHg or taking antihypertensive agent in subjects with a history of hypertension; (v) FPG level ≥ 100 mg/dL (5.6 mmol/L) or being put on drug treatment for elevated glucose.

### Statistical analysis

Statistical analysis was performed with the statistical package SPSS 20.0. The distribution of continuous variables was first tested using the Kolmogorov-Smirnov test. Data were presented as mean ± SD or median (interquartile ranges) depending on parameters with normal or non-normal distributions, and unpaired *t*-tests and Mann-Whitney *U* tests were adopted to compare these variables. A Chi-squared test was adopted for categorical variables. Spearman’s rank correlation coefficient was performed to analyze the correlations of fetuin-A, adiponectin and F/A ratio with other continuous variables of interest. Multivariate linear regression analysis with fetuin-A, adiponectin and F/A ratio as dependent variables was conducted to determine if confounding factors could influence their associations with the components of MS. Binary logistic regression analysis with MS as a dependent variable was applied to analyze its associations with fetuin-A, adiponectin and F/A ratio. When distributions of variables were generally non-normal, a logarithmic transformation was performed in regression analysis. Receiver operating characteristic (ROC) analysis curves were performed to evaluate and compare performances of fetuin-A, adiponectin and F/A ratio in identifying subjects with MS. A two-tailed *P* value of < 0.05 was considered statistically significant.

## Results

Clinical characteristics of all study subjects were summarized in Table 1. The number of subjects with and without MS was 284 and 181, respectively. The prevalence of MS in the elderly population included in this study was 38.9%. In addition to age and gender (*P* > 0.05), significant differences were observed in all the other listed anthropometric and metabolic parameters between subjects with and without MS (*P* < 0.001).

**Table 1:** The clinical characteristics of the study subjects based on the presence of metabolic syndrome

Parameters	Metabolic syndrome		*P*
	No (*n* = 284)	Yes (*n* = 181)	
Age (years)	65.93 ± 6.36	66.25 ± 6.51	0.292
Men, % (n)	147 (51.76)	102 (56.35)	0.333
Waist circumference (cm)	83.21 ± 8.47	90.68 ± 8.62	< 0.001
Body mass index (kg/m^2^)	24.18 ± 2.92	26.79 ± 3.05	< 0.001
Systolic blood pressure (mm Hg)	125.33 ± 18.21	139.17 ± 19.03	< 0.001
Diastolic blood pressure (mm Hg)	76.24 ± 11.18	87.39 ± 10.62	< 0.001
Fasting plasma glucose (mmol/l)	5.26 ± 0.77	6.05 ± 1.54	< 0.001
Total cholesterol (mmol/l)	4.89 ± 0.90	5.19 ± 0.99	< 0.001
Triglyceride (mmol/l)	1.32 ± 0.63	2.48 ± 1.19	< 0.001
HDL cholesterol (mmol/l)	1.49 ± 0.47	1.28 ± 0.53	< 0.001
LDL cholesterol (mmol/l)	2.38 ± 0.72	3.16 ± 0.89	< 0.001
HOMA-IR	2.25 (1.68, 2.95)	3.76 (2.77, 4.63)	< 0.001
C-reactive protein (mg/l)	1.85 (1.10, 2.69)	3.46 (2.48, 4.62)	< 0.001
Fetuin-A (mg/l)	251.85 (201.73, 292.70)	300.00 (254.80, 352.50)	< 0.001
Adiponectin (mg/l)	9.25 (8.30, 10.50)	8.00 (7.25, 9.10)	< 0.001
F/A ratio	28.34 (19.99, 34.99)	39.19 (30.47, 48.26)	< 0.001

Data are given as mean ± SD or median (interquartile ranges)

*HDL* high-density lipoprotein, *LDL* low-density lipoprotein, *HOMA-IR* homeostasis model assessment of insulin resistance, *F/A* fetuin-A/adiponectin

The Spearman’s rank correlation coefficients among fetuin-A, adiponectin, F/A ratio, and risk factors of MS were illustrated in Table 2. There was a significantly negative correlation between fetuin-A and adiponectin (*P* < 0.001). Fetuin-A was positively correlated with WC, FPG, TG, LDL-C, HOMA-IR and CRP; while adiponectin was negatively correlated with WC, DBP, FPG, TC and CRP, and positively with HDL-C. F/A ratio was significantly correlated with all the listed risk factors of MS except for BMI.

**Table 2:** Spearman’s correlation analysis among fetuin-A, adiponectin, F/A ratio and risk factors of metabolic syndrome

Parameters	Fetuin-A	Adiponectin	F/A ratio
Waist circumference	**0.393 (< 0.001)**	**− 0.132 (0.004)**	**0.356 (< 0.001)**
Body mass index	0.074 (0.111)	− 0.090 (0.052)	0.075 (0.107)
Systolic blood pressure	0.071 (0.128)	− 0.078 (0.092)	**0.112 (0.016)**
Diastolic blood pressure	0.076 (0.103)	**− 0.091 (0.049)**	**0.104 (0.025)**
Fasting plasma glucose	**0.125 (0.007)**	**− 0.170 (< 0.001)**	**0.185 (< 0.001)**
Total cholesterol	0.084 (0.069)	**− 0.189 (< 0.001)**	**0.134 (0.004)**
Triglyceride	**0.123 (0.008)**	− 0.069 (0.138)	**0.140 (0.002)**
HDL cholesterol	**−** 0.070 (0.134)	**0.136 (0.003)**	**− 0.098 (0.035)**
LDL cholesterol	**0.115 (0.013)**	− 0.065 (0.159)	**0.121 (0.009)**
HOMA-IR	**0.242 (< 0.001)**	− 0.085 (0.067)	**0.257 (< 0.001)**
C-reactive protein	**0.276 (< 0.001)**	**− 0.153 (0.001)**	**0.265 (< 0.001)**
Fetuin-A	–	**− 0.437 (< 0.001)**	**0.917 (< 0.001)**
Adiponectin	**− 0.437 (< 0.001)**	–	**− 0.719 (< 0.001)**
F/A ratio	**0.917 (< 0.001)**	**− 0.719 (< 0.001)**	–

Data are given as *r* (*P*)

*HDL* high-density lipoprotein, *LDL* low-density lipoprotein, *HOMA-IR* homeostasis model assessment of insulin resistance, *F/A* fetuin-A/adiponectin

In multivariate linear regression analysis, after adjustments for age, sex and BMI, Fetuin-A was independently associated with WC, FPG and TG. Adiponectin was independently associated with WC, DBP, FPG and HDL-C. F/A ratio was independently associated with all the components of MS (Table 3).

**Table 3:** Multivariate linear regression analysis with fetuin-A, adiponectin and F/A ratio as dependent variables and with the components of metabolic syndrome as independent variables

Parameters	Fetuin-A		Adiponectin		F/A ratio	
	*β*	*P*	*β*	*P*	*β*	*P*
Waist circumference	**0.197**	**< 0.001**	**− 0.106**	**0.022**	**0.216**	**< 0.001**
Systolic blood pressure	0.051	0.199	− 0.067	0.143	**0.129**	**0.003**
Diastolic blood pressure	0.071	0.089	**− 0.095**	**0.039**	**0.086**	**0.043**
Fasting plasma glucose	**0.149**	**0.001**	**− 0.195**	**< 0.001**	**0.199**	**< 0.001**
Triglyceride	**0.113**	**0.014**	− 0.064	0.166	**0.118**	**0.006**
HDL cholesterol	− 0.037	0.324	**− 0.110**	**0.017**	**− 0.100**	**0.033**

*HDL* high-density lipoprotein, *F/A* fetuin-A/adiponectin; adjusted for sex, age and body mass index

To evaluate associations of fetuin-A, adiponectin and F/A ratio with incident MS, the logistic regression analysis with MS as a dichotomous dependent variable was performed (Table 4). The unadjusted OR values (95% CI) for the associations of fetuin-A, adiponectin and F/A ratio with incident MS were 1.109 (1.030–1.194), 0.712 (0.631–0.806) and 1.608 (1.325–1.952), respectively. After adjustments for age, sex and BMI, the adjusted OR values (95% CI) for their associations were 1.100 (1.020–1.186), 0.760 (0.664–0.871) and 1.466 (1.189–1.808), respectively.

**Table 4:** The adjusted and unadjusted odds ratio (OR) and 95% confidence intervals (CI) for the associations between metabolic syndrome and fetuin-A, adiponectin and F/A ratio

Parameters	Metabolic syndrome			
	Unadjusted OR (95% CI)	*P*	Adjusted OR (95% CI)	*P*
Fetuin-A	1.109 (1.030–1.194)	0.006	1.100 (1.020–1.186)	0.013
Adiponectin	0.712 (0.631–0.806)	0.001	0.760 (0.664–0.871)	0.004
F/A ratio	1.608 (1.325–1.952)	< 0.001	1.466 (1.189–1.808)	< 0.001

*F/A*fetuin-A/adiponectin; adjusted for sex, age and body mass index

**Fig. 1:**
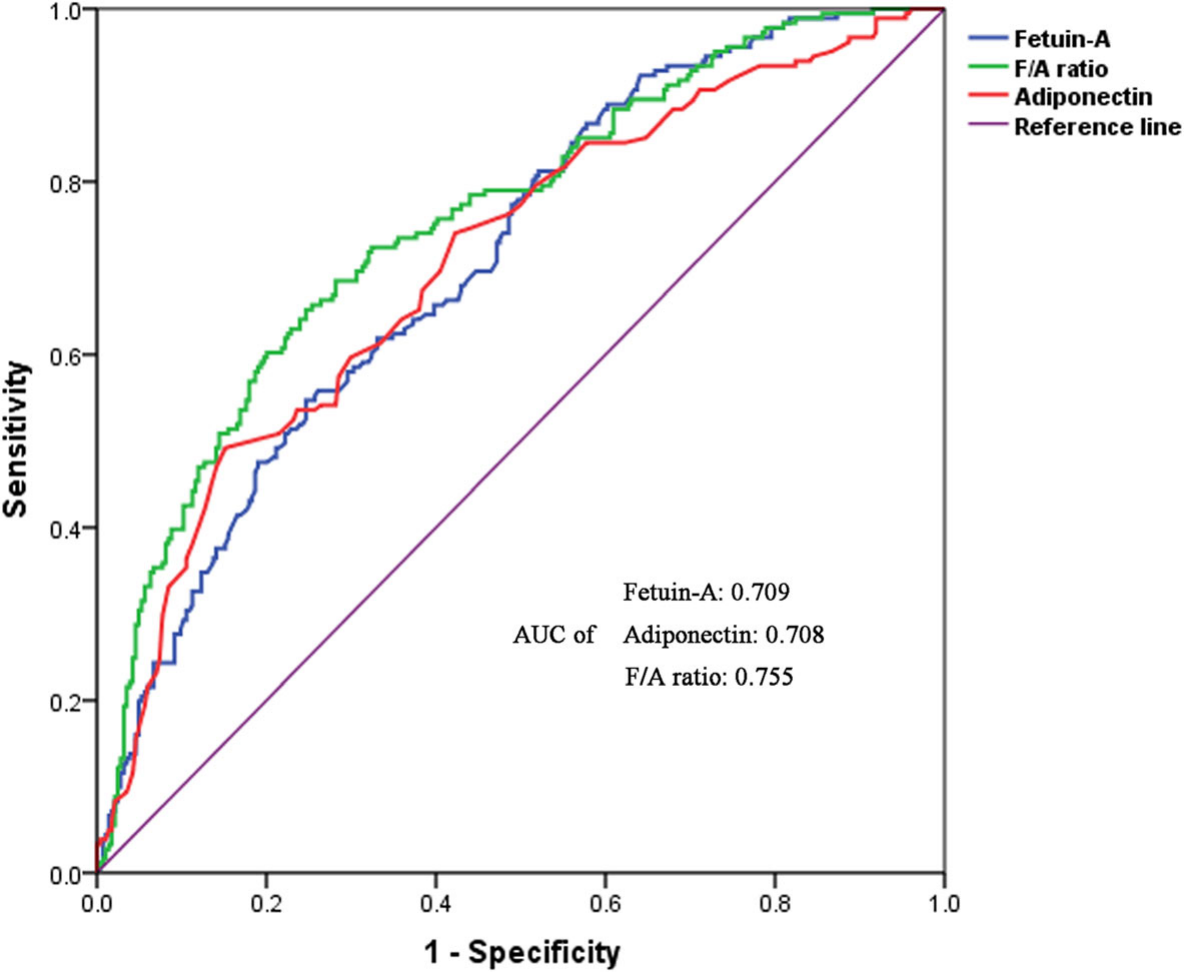
Receiver operating characteristic (ROC) analysis of fetuin-A, adiponectin and F/A ratio for identifying subjects with metabolic syndrome. F/A: fetuin-A/adiponectin

ROC analysis was performed to evaluate the diagnostic performance of fetuin-A, adiponectin and F/A ratio for MS (Fig. 1). AUC values of fetuin-A, adiponectin and F/A ratio for detecting MS were 0.709, 0.708 and 0.755, respectively (all *P* < 0.05). F/A ratio of 33.68 was identified as the best cut off value for diagnosing MS, with the corresponding sensitivity and specificity of 0.685 and 0.718, respectively.

## Discussion

In this study, we found that F/A ratio was significantly associated with all the components of MS and incident MS, and showed a better diagnostic performance in classifying subjects with and without MS than either fetuin-A or adiponectin. These findings suggest that F/A ratio may be a more sensitive indice for assessing MS than either of the two parameters in the elderly.

Systemic metabolic regulation is extremely complex and involves multiple organ and physiological actions, in which the liver and adipose tissue play key roles and interplay with each other [20]. Therefore, the choice indexes reflecting metabolic disorders should be a composite of several different indicators and take the liver and adipose tissue into consideration, which is often not available to some single biochemical parameters. Fetuin-A is produced mainly by liver cells, despite recent reports that it can also be produced and secreted by adipose tissue [4–6]; and adiponectin is secreted only by adipose cells [10]. In view of both fetuin-A and adiponectin involving in metabolic regulation and their opposite functions, their ratio may be a better indicator for examining metabolic disorders such as MS.

In fact, numerous studies have recently indicated that there is an interaction between fetuin-A and adiponectin. Agarwal et al. [21] have revealed that a lowering of circulating adiponectin levels is accompanied by higher fetuin-A levels in obese diabetic mice induced by high-fat diet, and have provided evidence that fetuin-A exerts its inhibitory effect on adiponectin through a Wnt3a-PPAR*γ* pathway. Adiponectin has been shown to ameliorate hepatic steatosis and the impairment of lipid metabolism in hepatocytes by suppressing the hepatokine fetuin-A via an AMPK pathway [22]. Ix et al. [23] have suggested that the elevated expression of fetuin-A and repressed expression of adiponectin in individuals with obesity and related complications exhibit cooperative effects of jointly inhibiting AMPK activation. Effective anti-obesity medications are significantly correlated with lower fetuin-A levels, higher adiponectin levels and AMPK stimulation. In an experiment which has explored the effects of resveratrol on metabolic regulation both in vivo and in vitro, a crosstalk between adiponectin and fetuin-A has been observed, and resveratrol-treated high-fat diet-induced obese mice have presented a decrease in serum fetuin-A levels and an increase in serum adiponectin levels [24]. Besides, in septic patients, decreased serum fetuin-A levels and increased adiponectin levels have been observed; and the study has pointed out that F/A ratio is a valuable marker for distinguishing the severity of sepsis and stratifying septic patients at risk [25]. Taken together, given the crosstalk between adiponectin and fetuin-A, F/A ratio has been recognized as a potentially promising marker for evaluating metabolic disorders and inflammation-related diseases.

Our findings are consistent with previous reports that there is a significant positive correlation between fetuin-A and MS and a significant negative correlation between adiponectin and MS [8, 9, 12–14]. In our study, F/A ratio presented a stronger association with MS. We also discovered a significant negative correlation between fetuin-A and adiponectin, which was also in conformity with the previous research [26]. We did not find the significantly respective correlations between fetuin-A and SBP, DBP and HDL-C and between adiponectin and SBP and TG. However, we observed that F/A ratio was significantly associated with all the components of MS even after adjustments for potential confounding factors. In the further ROC curve analysis, F/A ratio showed higher AUC, sensitivity and specificity values than either fetuin-A or adiponectin. These results further suggest that F/A ratio is a more sensitive indicator of MS than either of the two indices.

Besides, some limitations still exist in this study. Firstly, restricted by a cross-sectional design, the causal link between F/A ratio and MS cannot be established. Secondly, subjects included in this project are physical examination individuals rather than natural population, with the minimum age of 60. Thus, a potential selection bias in subjects is inevitable. Thirdly, what this study has measured is total adiponectin instead of high molecular weight adiponectin--a better predictor for evaluating MS and its components [27, 28]. The further investigation concerning roles of the ratio of fetuin-A to high molecular weight adiponectin in MS is requisite. Fourthly, some other potential confounders, such as eating habits, alcohol consumption and exercise, have not been collected in this study, which may have moderating effects on the relationship between F/A ratio and MS. Specifically, calorie restriction and exercise can result in decreased fetuin-A levels and increased adiponectin expressions [29, 30], and moderate alcohol consumption has been proven to be inversely associated with fetuin-A in men [31].

## Conclusions

In conclusion, the present study demonstrates that F/A ratio is a more sensitive indicator for evaluating MS than either fetuin-A or adiponectin in the elderly. Further well-designed trials with larger samples for corroborating the associations in natural population are expected.

## Data Availability

The datasets used and analyzed during the current study are available from the corresponding author on reasonable request.

## Abbreviations

MS: Metabolic syndrome
F/A: Fetuin-A/adiponectin
BMI: Body mass index
WC: Waist circumference
SBP: Systolic blood pressure
DBP: Diastolic blood pressure
FPG: Fasting plasma glucose
TC: Total cholesterol
TG: Triglyceride
HDL-C: High-density lipoprotein cholesterol
LDL-C: Low-density lipoprotein cholesterol
HOMA-IR: Homeostasis model assessment of insulin resistance
CRP: C-reactive protein
